# P2X7 receptor antagonists modulate experimental autoimmune neuritis via regulation of NLRP3 inflammasome activation and Th17 and Th1 cell differentiation

**DOI:** 10.1186/s12974-024-03057-z

**Published:** 2024-03-25

**Authors:** Yuhan Xie, Ranran Han, Yulin Li, Weiya Li, Shichao Zhang, Yu Wu, Yuexin Zhao, Rongrong Liu, Jie Wu, Wei Jiang, Xiuju Chen

**Affiliations:** 1 Department of Neurology, Tianjin Nankai Hospital, Tianjin Medical University, Tianjin, 300052 China; 2 Department of Neurology, Tianjin Neurological Institute, Tianjin Medical University General Hospital, Tianjin, 300052 China; 3 Department of Urology, Tianjin Institute of Urology, The Second Hospital of Tianjin Medical University, Tianjin, 300102 China; 4 Department of Radiology, The Third Affiliated Hospital of Zhengzhou University, Zhengzhou, Henan China

**Keywords:** Brilliant blue G, Guillain–Barré syndrome, CD4^+^ T cell, P2X7R

## Abstract

**Background:**

Guillain–Barré syndrome (GBS), a post-infectious, immune-mediated, acute demyelinating disease of the peripheral nerves and nerve roots, represents the most prevalent and severe acute paralyzing neuropathy. Purinergic P2X7 receptors (P2X7R) play a crucial role in central nervous system inflammation. However, little is known about their role in the immune-inflammatory response within the peripheral nervous system.

**Methods:**

Initially, we assessed the expression of purinergic P2X7R in the peripheral blood of patients with GBS using flow cytometry and qRT-PCR. Next, we explored the expression of P2 X7R in CD4^+^ T cells, CD8^+^ T cells, and macrophages within the sciatic nerves and spleens of rats using immunofluorescence labeling and flow cytometry. The P2X7R antagonist brilliant blue G (BBG) was employed to examine its therapeutic impact on rats with experimental autoimmune neuritis (EAN) induced by immunization with the P0_180 − 199_ peptide. We analyzed CD4^+^ T cell differentiation in splenic mononuclear cells using flow cytometry, assessed Th17 cell differentiation in the sciatic nerve through immunofluorescence staining, and examined the expression of pro-inflammatory cytokine mRNA using RT-PCR. Additionally, we performed protein blotting to assess the expression of P2X7R and NLRP3-related inflammatory proteins within the sciatic nerve. Lastly, we utilized flow cytometry and immunofluorescence labeling to examine the expression of NLRP3 on CD4^+^ T cells in rats with EAN.

**Results:**

P2X7R expression was elevated not only in the peripheral blood of patients with GBS but also in rats with EAN. In rats with EAN, inhibiting P2X7R with BBG alleviated neurological symptoms, reduced demyelination, decreased inflammatory cell infiltration of the peripheral nerves, and improved nerve conduction. BBG also limited the production of pro-inflammatory molecules, down-regulated the expression of P2X7R and NLRP3, and suppressed the differentiation of Th1 and Th17 cells, thus protecting against EAN. These effects collectively contribute to modifying the inflammatory environment and enhancing outcomes in EAN rats.

**Conclusions:**

Suppression of P2X7R relieved EAN manifestation by regulating CD4^+^ T cell differentiation and NLRP3 inflammasome activation. This finding underscores the potential significance of P2X7R as a target for anti-inflammatory treatments, advancing research and management of GBS.

**Supplementary Information:**

The online version contains supplementary material available at 10.1186/s12974-024-03057-z.

## Background

Guillain–Barré syndrome (GBS) is an acute inflammatory polyneurogenic disease that can lead to motor dysfunction, sensory impairment, autonomic dysfunction, and respiratory failure [1, 2]. Effective treatments for these ailments include plasma exchange and intravenous immunoglobulin infusion [3]. However, 20% of patients continue to experience severe sequelae, and 5% die despite receiving immunotherapy [4, 5]. The primary mechanism underlying disease development is molecular mimicry between microbial and neural antigens, resulting in an abnormal autoimmune response targeting peripheral nerves and their spinal roots [6].

Experimental autoimmune neuritis (EAN) mimics the clinical, histopathologic, and electrophysiologic features of GBS [7, 8]. Blood-nerve barrier breakdown is a characteristic feature of its pathophysiology, with the peripheral nervous system (PNS) being invaded by activated T cells and macrophages that demyelinate the peripheral nerves [9]. The cellular immune response plays a considerable role in the onset of GBS. Macrophages and CD4^+^ T cells enter the PNS and damage the nervous system either directly through phagocytic attack or indirectly through T cell-mediated cytotoxicity, as well as through cytokines and oxygen free radicals [10, 11]. Activated T lymphocytes may also promote B cells growth and differentiation into plasma cells, which secrete antibodies against the phospholipid components of the peripheral medulla [12]. The clinical scores of patients with GBS change in correlation with changes in CD4^+^ T-lymphocyte subsets [13, 14]. Regulating CD4^+^ T cells differentiations can alleviate EAN symptoms [14], but the endogenous molecular regulatory mechanism of CD4^+^ T differentiation remains unclear.

The nucleotide receptor family (P2 receptors, P2Rs) comprises two subfamilies: G-protein-coupled metabotropic P2Y (P2YR) and the ligand (ATP)-gated ionotropic P2X (P2XR) receptors [15]. P2X7R is a member of the P2X receptor subfamily of P2 receptors and is expressed in most innate and adaptive immune cells [16], including macrophages, monocytes, dendritic cells, and T cells [17, 18]. P2X7R is strongly correlated with inflammation and involved in inflammatory disorders, including inflammatory bowel disease [19], rheumatoid arthritis [20], acute pancreatitis-associated lung injury [21], and Alzheimer’s disease [22].

In this study, we used an EAN model to examine the role of P2X7R in neuroinflammation. We investigated the signaling pathways and molecular mechanisms underlying P2X7R-mediated immunomodulation in the PNS. Furthermore, we examined the anti-inflammatory effects of brilliant blue G (BBG), a safe and highly selective P2X7R antagonist [23–25], in EAN rats. We found that BBG reduced neuroinflammatory reactions and neurological impairments by regulating CD4^+^ T cell differentiation and NLRP3 inflammasome activation in an EAN rat model. These findings suggest that targeting CD4^+^ T cell P2X7R may assist in the treatment of GBS.

## Materials and methods

### Patients and healthy control group

Fifteen patients with GBS who met the criteria outlined were recruited from Tianjin Medical University General Hospital and Tianjin Nankai Hospital. The inclusion criteria were the onset of weakness within 2 weeks, accompanied by an inability to walk independently for a distance of 10 m GBS disability score > 3) [26, 27]. The exclusion criteria were under 18 years of age, a history of GBS, pregnancy, lactation, immunosuppressive therapy, antacid treatment, and severe concurrent complications [28, 29]. As controls, 15 age- and sex-matched healthy volunteers were included. Blood samples from patients with GBS (collected prior to the initiation of treatment with intravenous immunoglobulin, or plasmapheresis) and healthy controls were utilized for qRT-PCR and flow cytometry analyses (Additional file 1: Table S1). All samples were collected after obtaining informed consent and in accordance with the ethical guidelines of the Institutional Review Board at Tianjin Medical University General Hospital and Tianjin Nankai Hospital.

### Animals

Male Lewis rats (6–8 weeks-old; 160–190 g) were purchased from the Vital River Corporation, Beijing, China. The rats were housed under specific pathogen-free conditions with a 12-h light/dark cycle, given free access to food and water, and randomly divided into prophylactic, therapeutic, and vehicle groups.

### Induction of EAN and evaluation of clinical signs

A 300µL inoculum dose containing 300 µg of dissolved P0 peptide 180–199 (10 mg/mL; Bio-Synthesis) emulsified with an equivalent volume of complete Freund’s adjuvant (Sigma-Aldrich) with *Mycobacterium tuberculosis* H37RA (Becton, Dickinson and Company) at a final concentration of 1 mg/mL was administered to Lewis rats in both hind footpads.

Two investigators evaluated the rats’ neurological signs daily using the following scale: 0 (normal), 1 (reduced tail tonus), 2 (limp tail), 3 (absent righting), 4 (gait ataxia), 5 (mild hind limb paresis), 6 (moderate paraparesis), 7 (severe paraparesis or paraplegia of the hind limbs), 8 (tetraparesis), 9 (moribund), and 10 (death).

### BBG treatment

Lyophilized powder of BBG (50 mg/kg, purity > 95%; MedChemExpress) was diluted in saline. Diluted BBG solution was administered intraperitoneally to EAN rats as follows: to the prophylactic group, BBG was administered continuously from the first day of immunization (day 0) until the peak of morbidity (day 18); to the treatment group, BBG was administered from the first day when neurological signs were observed (day 8) until the peak of morbidity; and the vehicle group received an equivalent dose of saline.

### Histopathology

The rats from each group were anesthetized and perfused intracardially with cold PBS at the peak of the disease. Sciatic nerves were harvested and fixed in 4% paraformaldehyde overnight at 4 °C. The nerves were then dehydrated, vitrified, and embedded in paraffin. Transverse sections, 6 μm thick, were cut using a microtome (Leica RM2255) and stained with hematoxylin and eosin (Solarbio Science & Technology, China) and Luxol Fast Blue (LFB) (Abcam). Three random microscopic views were selected for sciatic nerve sections of each EAN rat and photographed using a Nikon microscope (200× magnification). Two independent observers (who were blinded to the treatment group) assessed inflammatory cell infiltration and demyelination. Inflammatory cell infiltration per square millimeter was quantified from three random microscopic views. Demyelinated lesions were identified as being stained with a lighter blue or remaining unstained under LFB staining at the same time. Histological scores were evaluated according to the following semi-quantitative pathological scale: 0 denoting a normal perivascular area; 1 indicating mild demyelination adjacent to the vessel; 2 representing moderate demyelination in proximity to the vessel; and 3 reflecting demyelination throughout the entire section.

### Electrophysiologic analysis

Electromyography of the sciatic nerve was performed in each group of EAN rats on day 18 after immunization using a fully digital KeyPoint Compact EMG/NCS/EP recording system (Dantec). Rats were anesthetized with chloral hydrate (3 mg/kg), and the sciatic nerve was exposed from the hip (proximal) to the ankle (distal). A pair of needle electrodes was inserted into the proximal sciatic nerve notch and distal ankle joint to stimulate evoked compound muscle action potentials (CMAPs), and the motor nerve conduction velocity (MNCV), amplitude, and latency of the CMAPs were recorded. We used 1 Hz pulses with an average power of 5 mA and a pulse width of 0.3 ms to stimulate the nerves and induce CMAPs. The recording electrodes were positioned in the “belly” part of the gastrocnemius muscle to record evoked potentials from stimulated sciatic nerve. MNCV was determined by measuring the separation between the stimulated cathodes and the resulting latency difference. Using the obtained CMAP curves, the amplitude was determined from the baseline to the maximum peak. Once electrophysiological tests were completed, the incision was stitched in an aseptic environment. We maintained body temperatures above 34 °C during electrophysiologic tests using heating pads under the animals. For each animal, measurements were performed in triplicate.

### Immunofluorescence staining

The sciatic nerves were fixed overnight in 4% paraformaldehyde at 4 °C, sequentially dehydrated in 15% and 30% sucrose, OCT-embedded, snap-frozen in liquid nitrogen, and then sectioned into 8-µm frozen slices using a cryostat (Leica Microsystems, Solms, Hessen, Germany). The sections were permeabilized with 0.3% Triton X-100 for 10 min and then washed. Non-specific binding sites were blocked with 3% BSA for 1 h. Next, the sections were incubated with the following antibodies overnight at 4 °C: rabbit anti-P2X7R (1:100, Alomone Labs), rabbit anti-IL-17 (1:200, Thermo Fisher Scientific, Waltham, MA, USA), mouse anti-CD4 (1:50, BioLegend), mouse anti-CD8 (1:50, BioLegend), mouse anti-CD68 (1:50, BioLegend), anti-NLRP3 (1:200, Thermo Fisher) and mouse anti-SOX10 (1:100, Thermo Fisher Scientific). Subsequently, the sections were washed with PBS and then incubated with Alexa Fluor 488-conjugated donkey anti-rabbit IgG (1:1000, Thermo Fisher Scientific) and Alexa Fluor 594-conjugated donkey anti-mouse IgG (1:1000; Thermo Fisher Scientific) at room temperature for 1 h. Following a PBS wash and covering with DAPI, images were captured using a fluorescence microscope (Nikon).

### Flow cytometry

After isolating peripheral blood samples from patients with GBS and healthy controls, peripheral blood mononuclear cells (PBMCs) were separated using density gradient centrifugation. Next, the PBMCs were stained with PerCP-Cyanine5.5 anti-human CD45 antibody (BioLegend), APC anti-human CD4 antibody (BioLegend), PE anti-human CD14 antibody (BioLegend), and PE-cy7 anti-human CD8 antibody (BioLegend) for 40 min at room temperature. Following fixation and permeabilization, the cells were further stained with rabbit anti-human P2X7R antibody (Boster Biological Technology) or rabbit anti-human NLRP3 antibody (Thermo Fisher) for 40 min at room temperature, followed by incubation with FITC-conjugated donkey anti-rabbit IgG antibody (Thermo Fisher Scientific) for an additional 40 min at room temperature.

Single-cell suspensions were prepared from the rat spleens at the peak of the neurological course. Splenic mononuclear cells (MNCs) were divided into three groups and then blocked with 1% BSA before antibody incubation. One group was stimulated with a 1× Cell Stimulation Cocktail along with protein transport inhibitors (Thermo Fisher) for 6 h. Cultures were harvested, incubated with APC anti-rat CD4 antibody (BioLegend) for 40 min at room temperature, and subsequently fixed and permeabilized. They were then stained with FITC anti-rat IFN-γ antibody (BioLegend), PE anti-rat IL-4 antibody (BioLegend), and PerCP-Cyanine5.5 anti-rat IL-17 A (Thermo Fisher) for 30 min at 4 °C in the dark.

The second group of Splenic MNCs underwent surface staining with APC anti-rat CD4 antibody (BioLegend) and PE anti-rat CD25 antibody (BioLegend) for 40 min at room temperature. After fixation and permeabilization using the eBioscience Foxp3/Transcription Factor Staining Buffer Set (Thermo Fisher Scientific), the cells were stained with anti-rat Alexa Fluor 488 Foxp3 antibody (BioLegend).

The third set of MNCs were subjected to surface staining with PerCP-Cyanine5.5 anti-rat CD3 antibody (BioLegend), APC anti-rat CD4 antibody (BioLegend), PE anti-rat CD8 antibody (BioLegend), and PE-cy7 anti-rat CD68 antibody (BioLegend) for 40 min at room temperature. Following fixation and permeabilization, the cells were then stained with rabbit anti-rat P2X7R antibody (Boster Biological Technology) or rabbit anti-rat NLRP3 antibody (Thermo Fisher) for an additional 40 min at room temperature. Finally, the cells were incubated with FITC-conjugated donkey anti-rabbit IgG antibody (Thermo Fisher Scientific) at room temperature for 40 min. All procedures were performed per manufacturer’s instructions. Flow cytometry was carried out using a BD Accuri Cflow (BD Biosciences, San Jose, CA, USA), and the acquired data were analyzed with FlowJo® version 10.0 software (Ashland, OR, USA).

### qRT-PCR

Total RNA was extracted from human PBMCs, EAN rat sciatic nerves and fresh rat splenic MNCs using TRIzol reagent (Glpbio, USA) according to the manufacturer’s guidelines. The cDNA was synthesized using TransScript First-Strand cDNA Synthesis Super Mix (TransGen Biotech) and qPCR was performed in triplicate using FastStart Universal SYBR Green Master Mix (Glpbio) on a CFX ConnectTM Real-Time PCR Detection System (Bio-Rad, USA). The primers used in the study are listed in Additional file 1: Table S2. The qRT-PCR steps including incubation at 95 °C for 10 min, followed by 40 cycles at 95 °C for 15 s and 60 °C for 1 min and was performed in duplicate. Relative gene expression was normalized to β-actin gene expression and calculated using the 2^−ΔΔCt^ method.

### Western blot analysis

Sciatic nerves were lysed in RIPA buffer (Solarbio) containing a protein phosphatase inhibitor cocktail. Proteins were separated by performing sodium dodecyl sulfate-polyacrylamide gel electrophoresis using 10% resolving gels and then transferred onto polyvinylidene difluoride membranes (Millipore, USA). The membranes were blocked with 5% non-fat milk and then incubated with the following primary antibodies overnight at 4 °C: anti-P2X7R (1:1000, Abcam), anti-NLRP3 (1:500, Thermo Fisher), anti-IL-1β (1:1000, Abcam), and anti-Caspase-1 (1:1000, Abcam). Next, the membranes were washed and incubated with donkey anti-rabbit IgG or donkey anti-mouse IgG conjugated with HRP at a 1:5000 dilution (Thermo Fisher Scientific) for 1 h at room temperature. Finally, the immunoreactive protein bands were detected using a Gel Doc imaging system (Bio-Rad), band intensity was expressed as the fraction corresponding to GAPDH and analyzed using ImageJ software.

### Statistical analysis

Data were analyzed using GraphPad Prism (GraphPad Software, Version 9.4; La Jolla, CA, USA). Differences between the clinical scores were analyzed using a two-way ANOVA. The Mann–Whitney U test was used to compare the differences between the two groups. One-way ANOVA (Kruskal–Wallis test) followed by Dunnett’s multiple-comparison test was used to analyze the differences among multiple groups. Data are shown as mean ± SEM. Results with *p <* 0.05 were considered statistically significant.

## Results

### P2X7R expression increased on CD4^+^ T cells obtained from patients with GBS

**Fig. 1:**
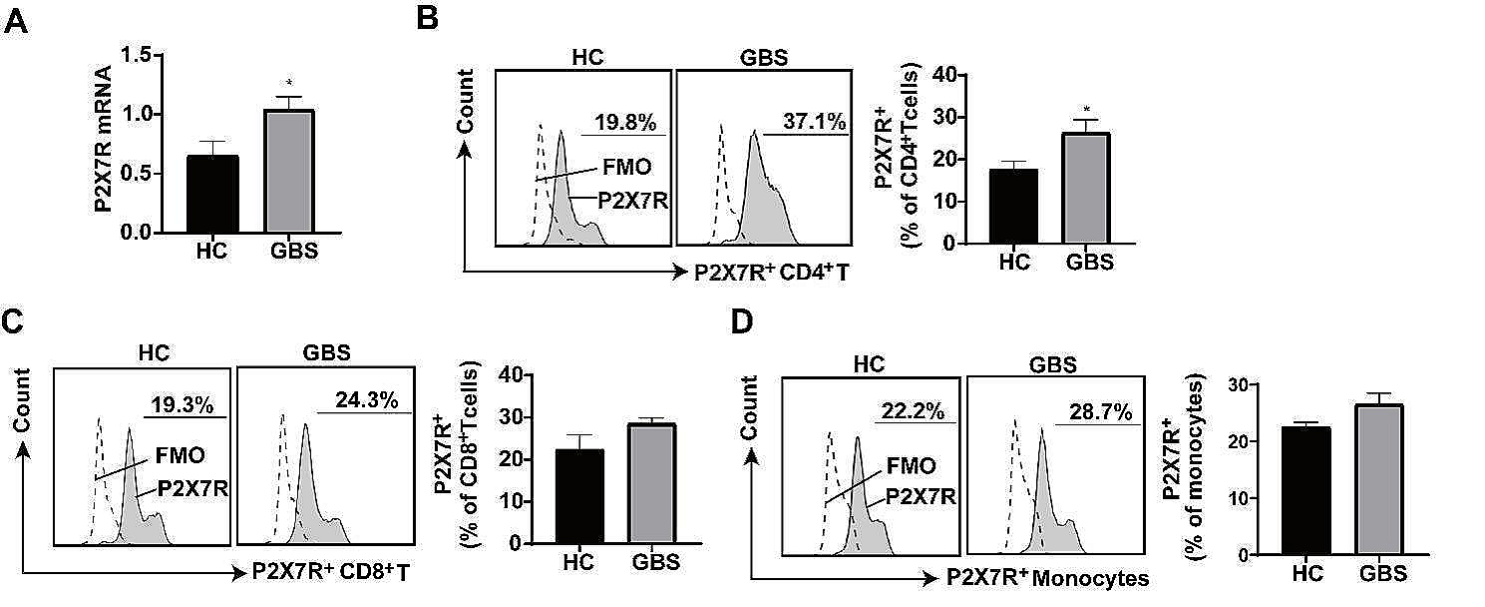
P2X7R expression in blood of patients with GBS and healthy controls. Human peripheral blood mononuclear cells (PBMCs) were isolated from blood samples, and P2X7R expression was examined using flow cytometry and qRT-PCR. **(A)** P2X7R mRNA levels were elevated in the PBMCs collected from patients with GBS compared to those in PBMCs collected from the healthy controls (*n* = 8 per group). **(B-D)** P2X7R expression increased on CD4^+^ T cells in the PBMCs collected from patients with GBS, but no significant increase was noted on CD8^+^ T cells or monocytes (CD14^+^) compared to healthy controls (*n* = 7 per group). HC, healthy control; GBS, Guillain–Barre Syndrome. *p < 0.05, **p < 0.01

Utilizing flow cytometry and qRT-PCR, we examined P2X7R expression on peripheral blood immune cells obtained from patients with GBS (Fig. 1). Patients with GBS exhibited upregulation of P2X7R mRNA levels on PBMCs compared to healthy controls (Fig. 1A). To further investigate the alteration of P2X7R on immune cells, FACS analysis was performed to evaluate its expression on different immune cells (gating strategy in Additional file 1: Fig. S3A). We observed increased P2X7R expression on CD4^+^ T cells (Fig. 1B), while CD8^+^ T cells and monocytes showed non-significant increases in P2X7R expression in patients with GBS compared to those in healthy controls (Fig. 1C-D). These results indicated that P2X7R expression increased in immune cells in patients with GBS, especially in CD4^+^T cells.

### P2X7R expression significantly increased on CD4^+^ T cells obtained from EAN rats

**Fig. 2:**
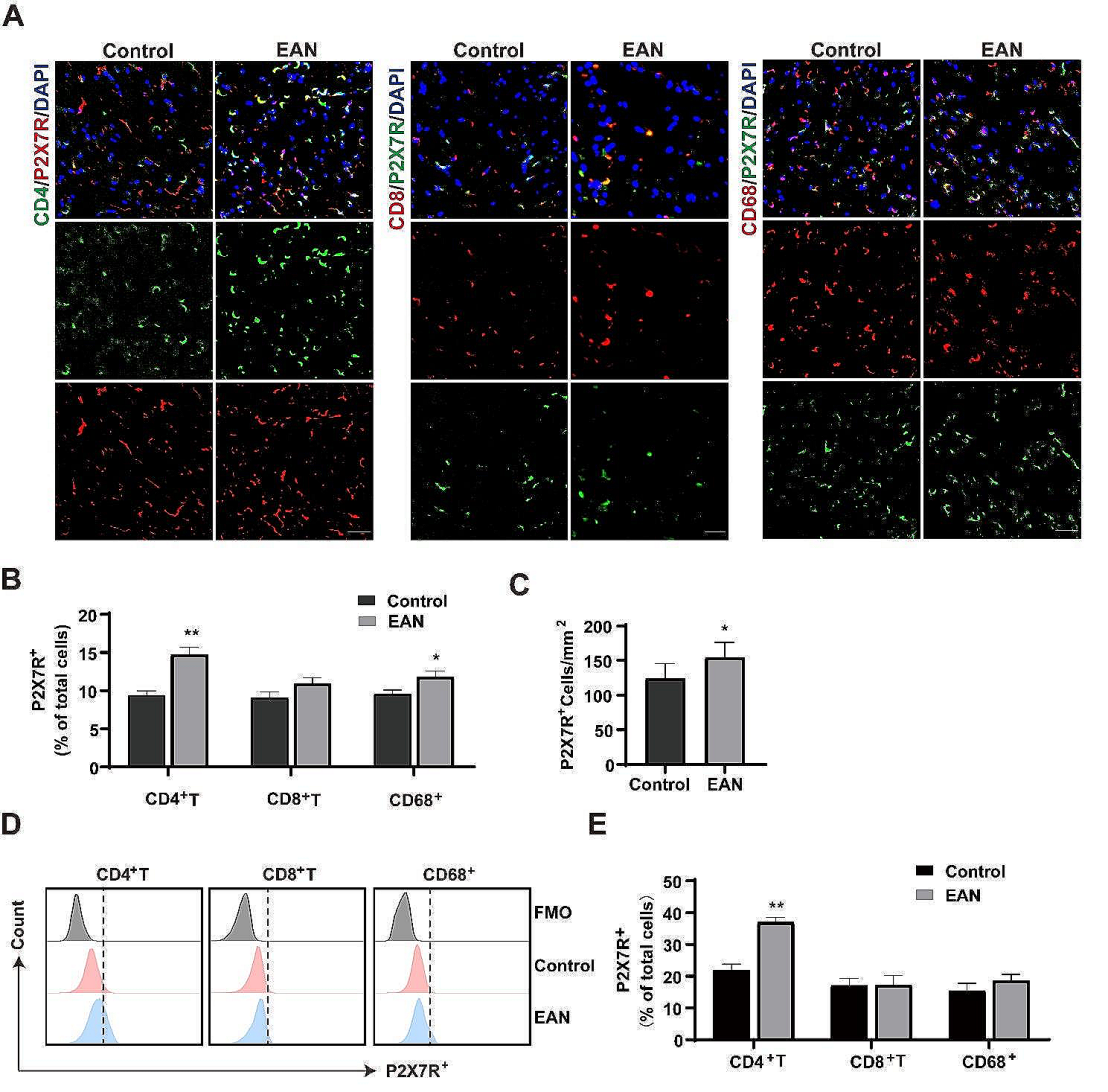
P2X7R expression was significantly elevated in CD4^+^ T cells in EAN rats. P2X7R expression in T cells and macrophages in control (unimmunized group) and EAN rats at day 18. **(A-B)** Fluorescence photomicrographs showing P2X7R expression and quantification in CD4^+^ T cells, CD8^+^ T cells and macrophages (CD68^+^) in the sciatic nerves of EAN and control rats. Scale bars, 20 μm. **(C)** Graph showing the number of P2X7R-positive cells in sciatic nerve sections obtained from control and EAN rats. **(D-E)** Flow cytometry analysis of P2X7R expression and quantification on CD4^+^ T cells, CD8^+^ T cells and macrophages in the spleen of EAN and control rats. Experiments were performed in triplicate (*n* = 6 per replicate) with similar results. *p < 0.05, **p < 0.01

We used flow cytometry and immunofluorescence double labeling of CD4^+^ T cells, CD8^+^ T cells, and macrophages in sciatic nerves of EAN rats to confirm the extent of P2X7R expression (Fig. 2). In the sciatic nerves of the EAN group compared to the control group (unimmunized group), the expression of P2X7R increased significantly on CD4^+^ T cells (Fig. 2A-B). The immunofluorescence results showed an augmented presence of P2X7R^+^ cells per square millimeter (Fig. 2C). FCAS analysis was performed on splenic MNCs (gating strategy in Additional file 1: Fig. S4A), which also showed an increase of P2X7R expression on CD4^+^ T cells (Fig. 2D-E). It suggests that P2X7R in CD4^+^ T cells may play an important role in mediating neuritis pathogenesis in EAN.

### Inhibiting P2X7R with antagonist attenuated the neurological symptoms of EAN

**Fig. 3:**
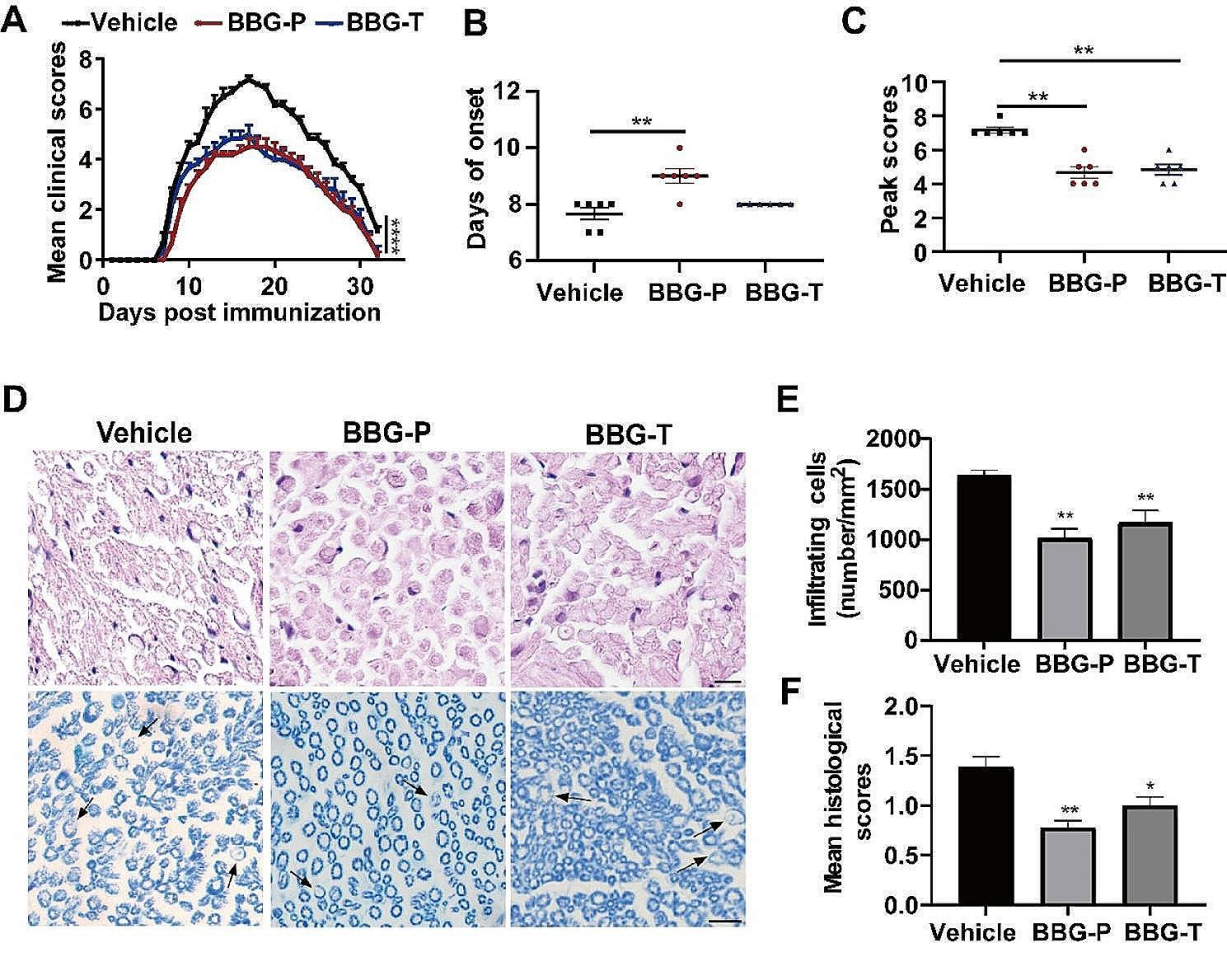
BBG treatment attenuated P0_180 − 199_ induced EAN. P0_180 − 199_ in complete Freund’s adjuvant was injected into Lewis rats (day 0) to immunize them. **(A)** Neurological function scores of rats in the prevention, treatment, and vehicle groups from day 7 to day 28 post-injection. **(B-C)** BBG treatment delays onset in the prevention group of EAN rats and lowers the maximum scores in EAN rats. **(D)** Hematoxylin and eosin staining and LFB staining of EAN rat sciatic nerve were performed to assess inflammatory cell infiltration and demyelination, respectively Demyelination is indicated by arrows; the demyelinated lesion is lighter or absent of blue staining. Representative photomicrographs are shown for each group. Scale bars, 10 μm. **(E)** Mean number of inflammatory cells per square millimeter of tissue section. **(F)** Degree of demyelination, as indicated by the histological scores. Experiments were performed in triplicate (*n* = 6 per replicate) with similar results. BBG-P, preventative BBG group; BBG-T, therapeutic BBG group. *p < 0.05, **p < 0.01

P2X7R was inhibited using BBG in two different strategies to treat the EAN in rat model (Fig. 3). BBG injection was delivered either before immunization for disease prevention or after immunization for treatment, and the rats were monitored daily for neurological symptoms. Both the preventative and therapeutic groups exhibited lower EAN severity compared to the vehicle group. Neurological scores from onset were significantly alleviated in both the preventive and treatment groups compared with those in the vehicle group (Fig. 3A). The vehicle group experienced EAN onset on the eighth day following vaccination, while the prophylactic group exhibited onset on the ninth day (Fig. 3B). Peak neurological status scores were lower in the preventive group (median score 4.5) and the treatment group (median score 5) than in the vehicle group (median score 7.0) (Fig. 3C). We utilized hematoxylin and eosin staining to assess inflammatory cell infiltration and LFB labeling to indicate demyelination of the sciatic nerve at the peak of EAN onset, determining whether the reduction in neurological signs was associated with these processes (Fig. 3D). In comparison to the vehicle group, both the preventive and treatment groups exhibited significantly reduced inflammatory cell infiltration per square millimeter (Fig. 3E) and lower histological scores (Fig. 3F), milder demyelination.

### P2X7R inhibition improved EAN-induced peripheral nerve conduction impairment

**Fig. 4:**
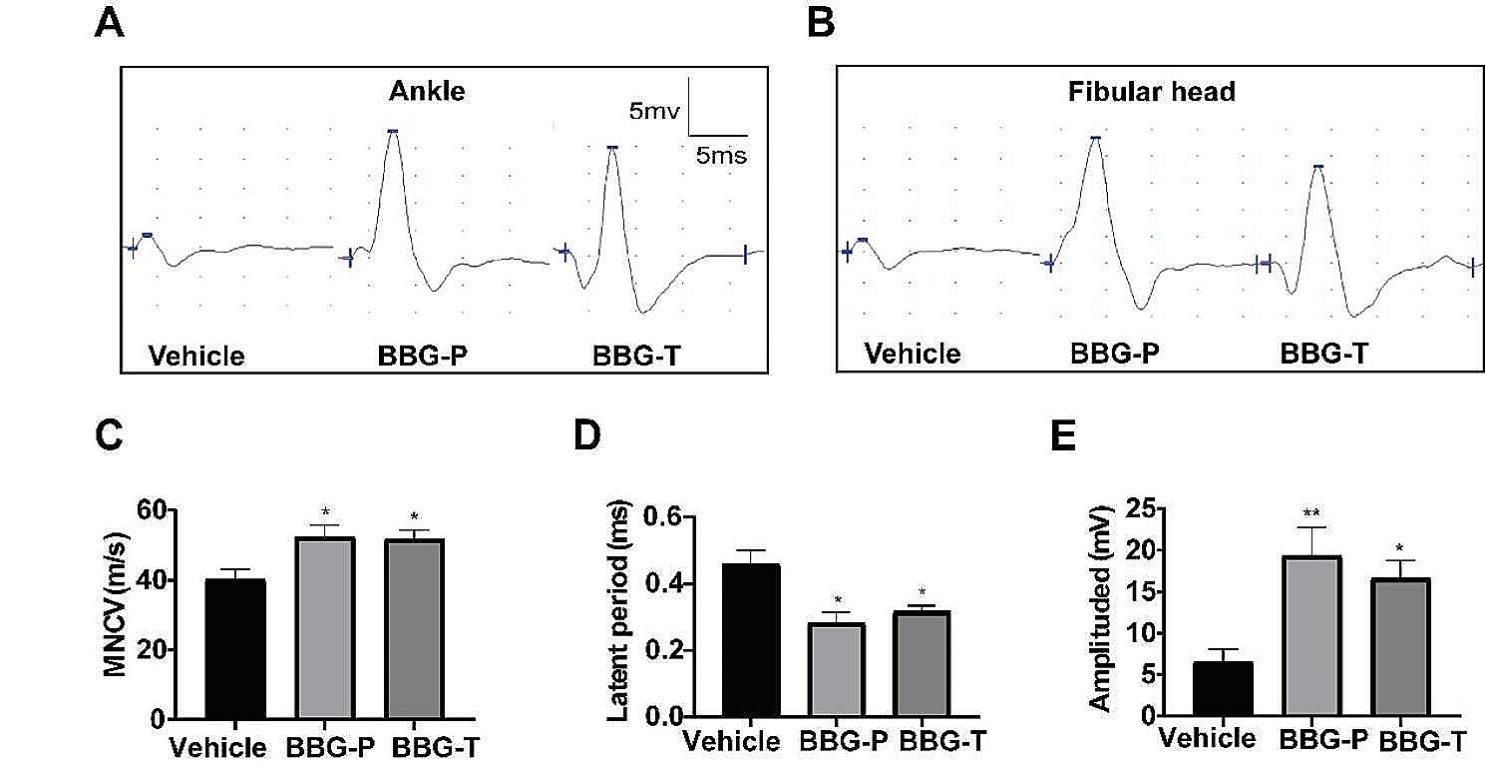
BBG attenuates peripheral nerves conduction damage in EAN. Compound muscle action potentials (CMAPs) were evoked by stimulation at the ankle joint **(A)** and fibular head **(B)**, and motor nerve conduction velocity (MNCV) **(C)**, latency **(D)**, and amplitude **(E)** of CMAPs were recorded. Experiments were performed in triplicate (*n* = 6 per replicate) with similar results. BBG-P, preventative BBG group; BBG-T, therapeutic BBG group. *p < 0.05, **p < 0.01

CMAPs were elicited at the ankle (distal) (Fig. 4A) and fibular head (proximal) (Fig. 4B) at the peak of EAN onset. Motor nerve conduction velocity (MNCV), amplitude, and latency were measured to assess the severity of peripheral nerve damage. P2X7R using BBG in prophylactic or treatment strategies mitigated the severity of peripheral nerve deficits. MNCV was faster in the prevention and treatment groups compared to that in the vehicle group (Fig. 4C). CMAP latency was shorter in the P2X7R-inhibited group compared to that in the vehicle group (Fig. 4D). Amplitude was higher in the P2X7R antagonist group than in the vehicle group (Fig. 4E).

### P2X7R inhibition suppressed Th1 and Th17 differentiation in EAN rats

**Fig. 5:**
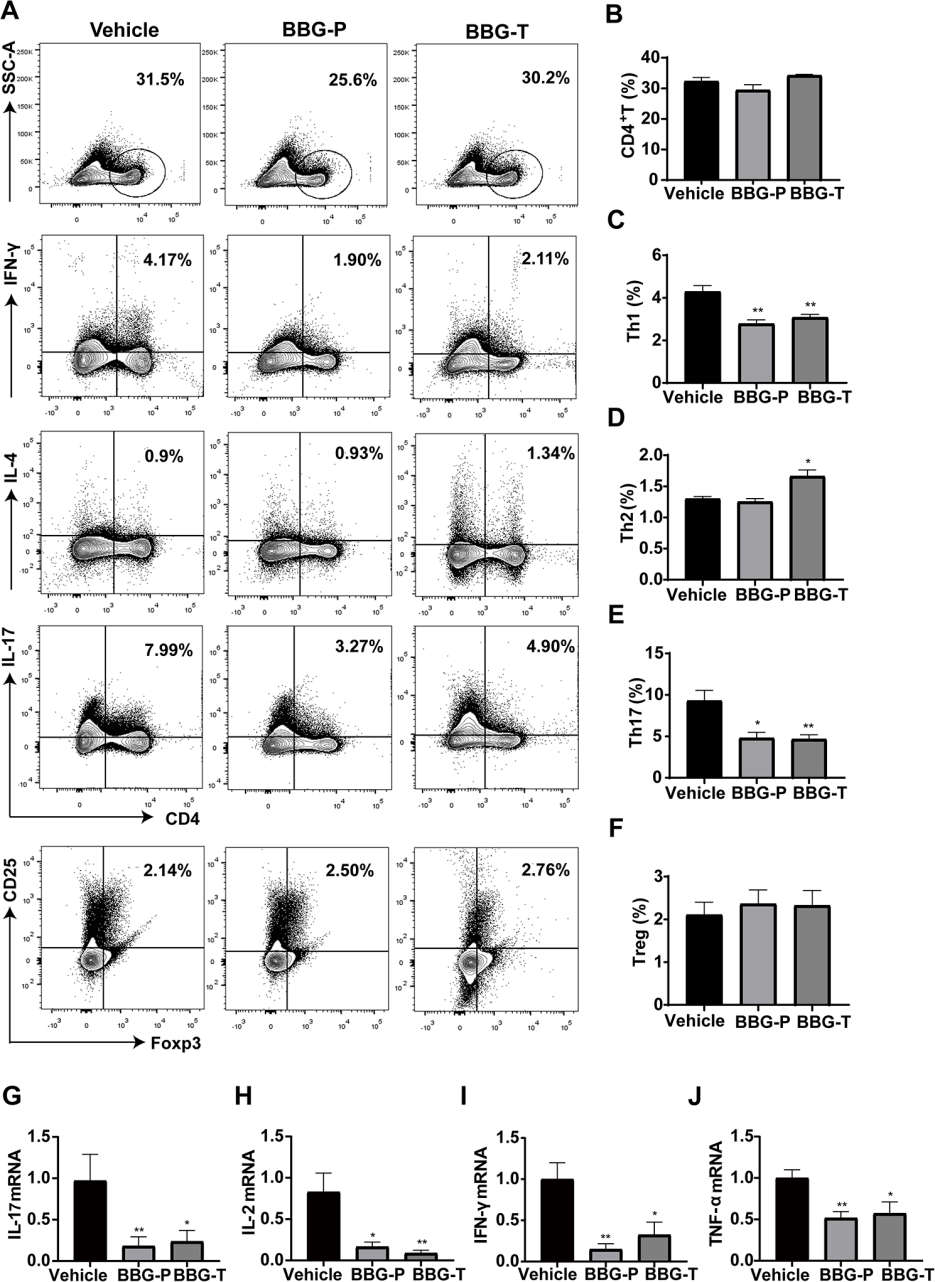
BBG suppressed Th1 and Th17 differentiation and reduced the expression of inflammatory cytokines in the spleen of EAN rats. EAN rats were sacrificed at day 18, and splenic MNCs of EAN rats were isolated for the assessing CD4^+^ T cell subpopulations using flow cytometry. The expression of inflammatory factors was determined through qRT-PCR. **(A)** Percentage of CD4^+^T cells, Th1 CD4^+^ IFN-γ^+^ cells, Th2 CD4^+^ IL-4^+^ cells, Th17 CD4^+^ IL-17^+^ cells and Treg CD4^+^ CD25^+^ Foxp3^+^ cells for each group. **(C, E)** Proportions of Th1 and Th17 cells among CD4^+^T cells decreased in the BBG-treated group compared to those in the vehicle group. **(B, D, F)** Proportions of splenic mononuclear CD4^+^T cells, and those of Th2 and Treg cells among CD4^+^T cells no effect. **(G-J)** mRNA levels of inflammatory cytokines IL-17, IL-2, IFN-γ, and TNF-α in splenic monocytes. Experiments were performed in triplicate (*n* = 6 per replicate) with similar results. BBG-P, preventative BBG group; BBG-T, therapeutic BBG group. *p < 0.05, **p < 0.01

**Fig. 6:**
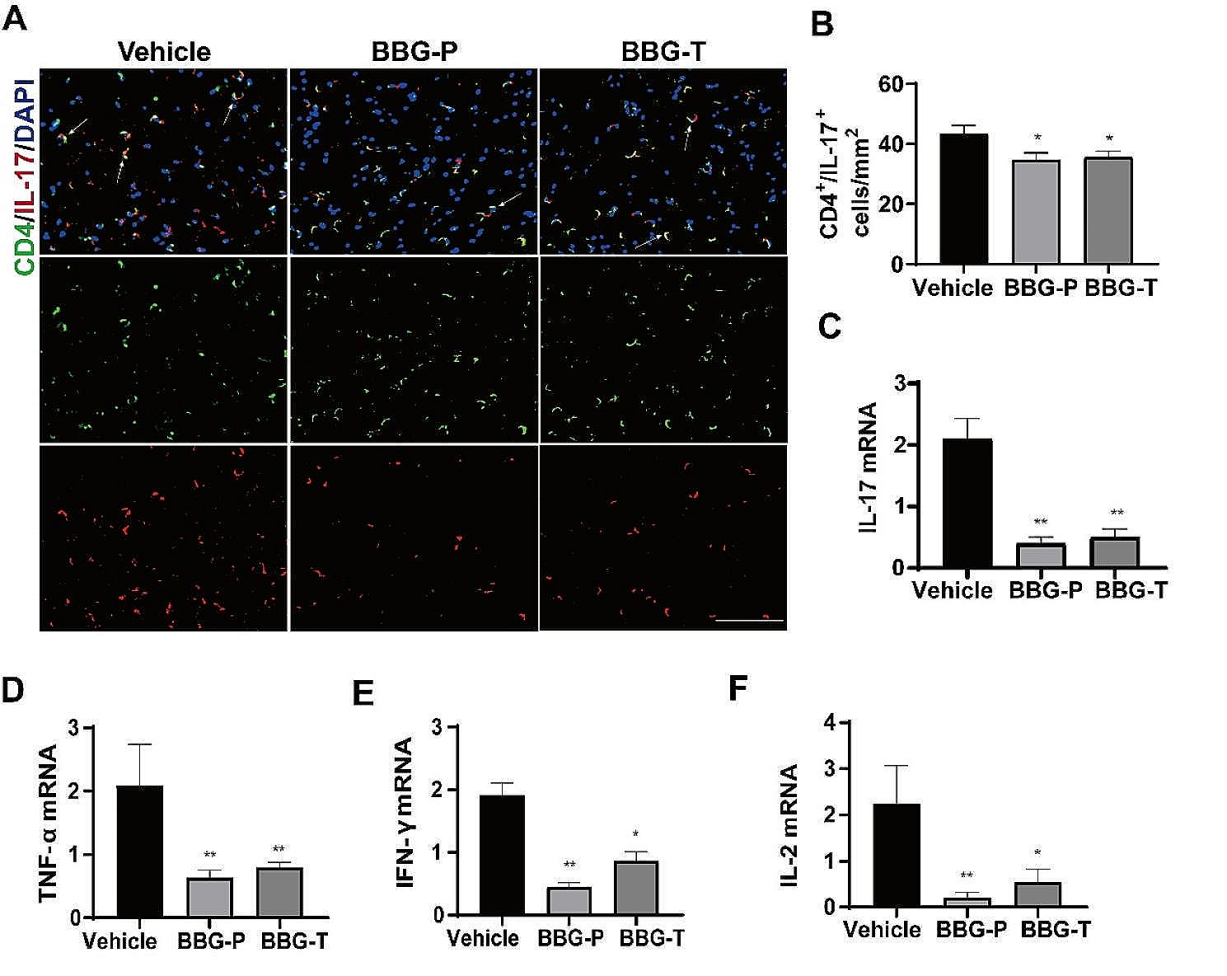
BBG reduced Th17 cell infiltration and the expression of inflammatory cytokines in the sciatic nerve of EAN rats. Sciatic nerves were harvested for immunofluorescence staining and qRT-PCR at the peak of EAN. **(A)** Fluorescence photomicrographs showing Th17 cells (arrows) in the sciatic nerves of EAN rats. Scale bars, 50 μm. **(B)** Th17 cells were quantified using by ImageJ software. Numbers of Th17 cells per square millimeter in the sciatic nerves. **(C-F)** mRNA levels of inflammatory cytokines IL-17, TNF-α, IFN-γ, and IL-2 in the sciatic nerve. Experiments were performed in triplicate (*n* = 6 per replicate) with similar results. BBG-P, preventative BBG group; BBG-T, therapeutic BBG group. *p < 0.05, **p < 0.01

To further investigate the potential neuroprotective mechanisms of P2X7R inhibition by BBG, we isolated rat splenic MNCs at the peak of EAN pathogenesis and subjected them for flow cytometric analysis (Fig. 5). As P2X7R expression mainly increased in CD4^+^ T cells, the differentiation of Th1, Th2, Th17 and Treg cells was evaluated. The proportion of Th1 and Th17 cells was significantly lower in the prevention and therapy groups than in the vehicle group. (Figure 5C and E). Compared with those in the vehicle group, the proportions of CD4^+^ T lymphocytes, Tregs, and Th2 cells were not significantly altered in the prevention or therapy groups (Fig. 5B, D and F). The proportion of Th17 cells was also lower in the prevention and therapy groups than that in the vehicle group, as evidenced by double immunofluorescence labeling of the sciatic nerve (Fig. 6A-B). These findings suggest that P2 × 7R inhibition by BBG may exert neuroprotective effects by regulating CD4^+^ T cell differentiation.

### Inhibition of P2X7R reduced the expression of pro-inflammatory cytokines

Changes in expressions of mRNAs encoding cytokines were determined in the immune microenvironment of the peripheral nerve by performing qRT-PCR. The mRNA levels of IL-17, IFN-γ, TNF-α, and IL-2 were downregulated in both the prevention and therapy groups compared to those in the vehicle group in EAN rats. This analysis was both performed using splenic MNCs (Fig. 5G-J) and the sciatic nerves (Fig. 6C-F) at the peak of the disease. These findings imply that blocking P2X7R with BBG suppressed the expression of pro-inflammatory molecules and protected rats from EAN.

### P2X7R inhibition suppressed NLRP3, IL-1β, and Caspase-1 protein levels in EAN rats

**Fig. 7:**
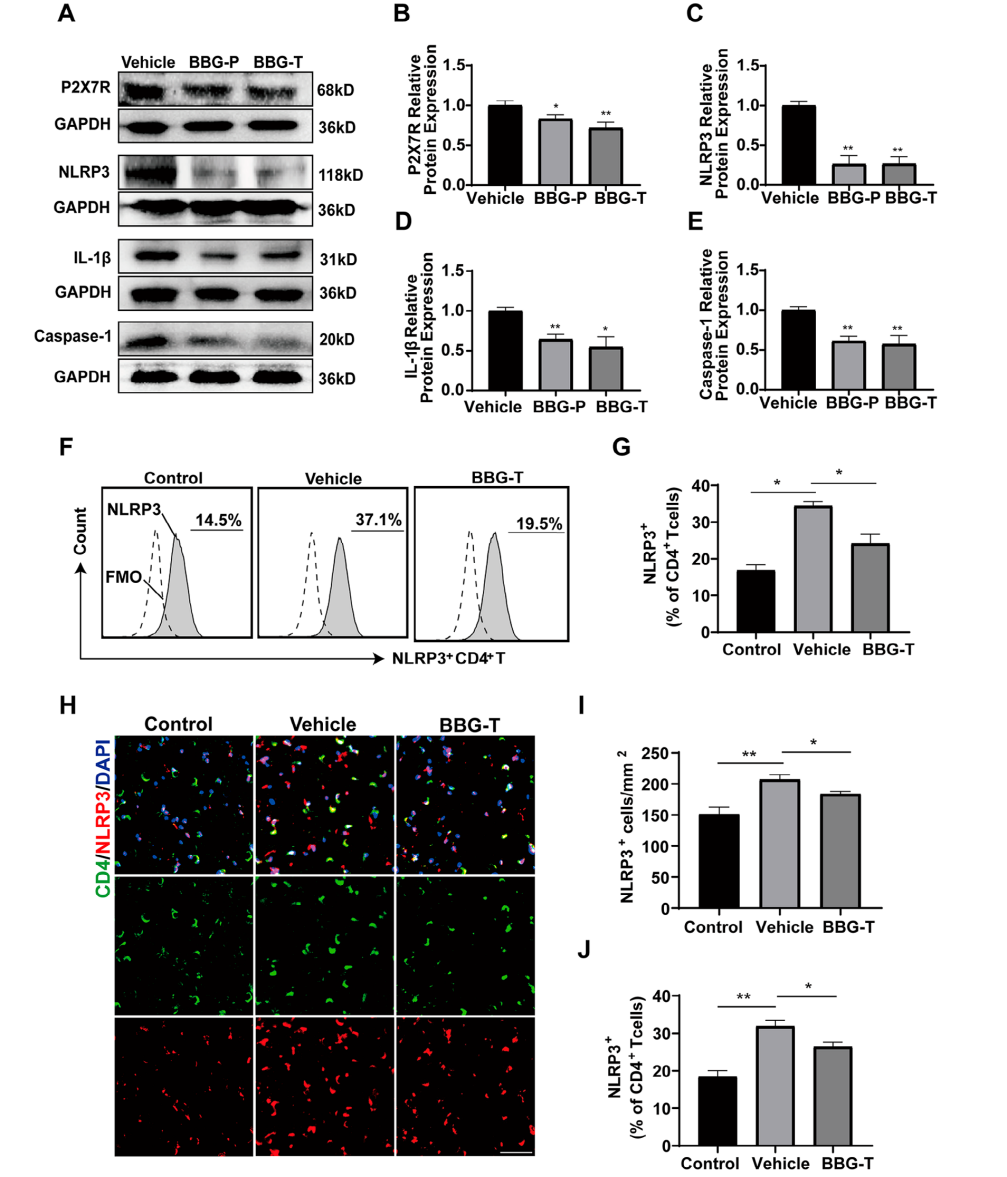
BBG treatment suppressed the P2X7R/NLRP3 pathway in CD4^+^T cells in EAN. Proteins were obtained from sciatic nerves at the peak of EAN and subjected to western blot analysis and quantification. NLRP3 expression in immune cells was analyzed through flow cytometry and immunofluorescence. **(A)** Representative western blots of P2X7R, NLRP3, IL-1β and Caspase-1 protein expression in sciatic nerve. **(B-E)** Protein levels were quantified via densitometric analysis of immunoreactive bands in western blots using ImageJ software and were normalized to that of GAPDH. **(F-G)** Flow cytometry analysis and NLRP3 quantification on CD4^+^ T cells in splenic MNCs of control (unimmunized group), vehicle and BBG-treated rats. **(H)** Immunofluorescence photomicrographs of NLRP3 expression in CD4^+^T cells in the sciatic nerves of control, vehicle, and BBG-treated EAN rats. **(I)** Number of NLRP3-positive cells in sciatic nerve sections obtained from control rats, vehicle and BBG-treated EAN rats. **(J)** NLRP3 quantification on CD4^+^T cells. Scale bars, 20 μm. Experiments were performed in triplicate (*n* = 6 per replicate) with similar results. Control group, unimmunized group; BBG-P, preventative BBG group; BBG-T, therapeutic BBG group. *p < 0.05, **p < 0.01

Next we explored the potential mechanism underlying the effect of P2X7R inhibition on modulating CD4^+^ T cell differentiation and cytokine production. We conducted western blot analyses of P2X7R and its downstream proteins using the sciatic nerves of EAN rats at the peak of disease onset (Fig. 7A). The results showed that the protein levels of P2X7R in sciatic nerve were reduced in the prevention and treatment groups compared to those in the vehicle group (Fig. 7B). Moreover, compared to the vehicle group, the preventative and therapy groups exhibited lower expression of NLRP3, IL-1β, and caspase-1 in the sciatic nerves of EAN. (Fig. 7C-E). To further clarify the downstream mechanism of P2X7R inhibition on CD4^+^T cells, we conducted flow cytometry analysis (gating strategy in Additional file 1: Fig. S4B) and immunofluorescence labeling to examine NLRP3 expression on CD4^+^ T cells. Compared with the control group, the vehicle group exhibited elevated expression whereas the BBG treatment group exhibited reduced expression of NLRP3 on splenic CD4^+^ T cells of EAN rats (Fig. 7F, G). An elevation of NLRP3 on PBMC CD4^+^ T cells was also confirmed in patients with GBS (Additional file 1: Fig. S1, gating strategy in Additional file 1: Fig. S3B). The immunofluorescence staining also revealed an upregulation of NLRP3 in sciatic nerve CD4^+^T cells (Fig. 7H-J). Collectively, these results revealed that P2X7R inhibition possibly exerts anti-inflammatory effects through the P2X7R/NLRP3 pathway in CD4^+^T cells in EAN rats.

## Discussion

In our investigation with a highly selective P2X7R antagonist BBG, the anti-inflammatory effect was identified as a previously unknown function of P2X7R inhibition in EAN, a classical animal model for GBS. Our research indicates that blocking P2X7R with BBG reduces neurological impairment and neuroinflammation in EAN. BBG-led P2X7R modulation influences the differentiation of Th1 and Th17 subsets of CD4^+^ T lymphocytes and suppresses NLRP3 inflammasome-mediated inflammatory responses. These results underscore the crucial neuroprotective role of P2X7R in the EAN model.

Both prophylactic and therapeutic BBG treatments reduced the severity of EAN symptoms, while prophylactic administration also delayed the onset of clinical symptoms, as determined by clinical scores. Pathologically, demyelination and inflammatory cell infiltration are hallmarks of EAN [8]. Compared to the vehicle group, the BBG-treated group exhibited significantly less inflammatory cell infiltration and demyelination in the sciatic nerve. Peripheral nerve damage in EAN results in decreased MNCV, prolonged CMAP latency, and reduced CMAP amplitude [30]. Our electrophysiologic findings demonstrate that MNCV, CMAP amplitudes, and latencies improved in the BBG-treated group, suggesting that BBG-mediated P2X7R inhibition enhances nerve conduction function. The release of cytokines associated with inflammatory vesicles is closely linked to P2X7R activation and tissue inflammation onset [31, 32]. Research studies have suggested a strong connection between IL-1β secretion and P2X7R inhibition [33]. Consistent with previous research, rats treated with BBG exhibit lower IL-1β expression in their sciatic nerves [34–36].

We observed a significant elevation in P2X7R levels in patients with GBS, primarily on CD4^+^ T cells. We also found compelling evidence of a substantial increase in P2X7R expression on CD4^+^ T cells in the EAN model. In comparison to CD8^+^ T cells and macrophages, we identified the most significant upregulation of P2X7R expression in CD4^+^ T cells of the EAN model, indicating that CD4^+^ T cell P2X7R is strongly associated with improved outcomes in EAN rats treated with BBG. Spleen, the largest peripheral immune organ in the body, serves as a site for the settlement of mature T lymphocytes [37], and the mobilization of splenic T cells and migration toward peripheral nerves may occur in EAN [38, 39]. To further explore the immune modulation effects of BBG after EAN, we investigate both sciatic nerves and spleens to detect the change of CD4^+^T cells subtype and inflammatory cytokine level. Flow cytometry data of spleen MNCs demonstrated a correlation between EAN improvement and a decrease in Th1 and Th17 CD4^+^ T cell subsets. A reduction of Th17 cells infiltration was also observed in sciatic nerves in the group of rats treated with BBG. Th cells play a crucial role in the pathophysiology of EAN [14, 40]. Th1 cells and Th1 cytokines, including IFN-γ and TNF-α, are considered the primary mediators of EAN [41]. IFN-γ promotes T cell differentiation towards a Th1 phenotype and suppresses the growth of Th2 cells in GBS, shifting the immunological response to a Th1 phenotype [42]. Moreover, GBS severity is correlated with serum TNF-α levels [43, 44], which damage peripheral myelin sheaths and the blood-nerve barrier, and promote pro-inflammatory Th cell growth [45]. Additionally, IL-17 A and Th17 cells may contribute to the EAN onset [46–48]. In the present study, we found a reduction of mRNA levels of IL-2, IL-17, IFN-γ, and TNF-α in spleens which is consistent with the changes in sciatic nerve. These findings support the hypothesis that BBG reduces EAN by inhibiting the development of Th1 and Th17 cells.

P2X7R controls various cellular signaling pathways, such as the release of cytokines and chemokines, activation of the NLRP3 inflammasome, cell death, and autophagy [49]. Of these pathways, the P2X7R-NLRP3 signaling pathway, has been associated with cognitive deficits in several neurological conditions, including Alzheimer’s disease [50], vascular cognitive impairment [51], and diabetes mellitus [52]. However, its role in inflammatory neuropathies like GBS remains uncertain. P2X7R activation can reportedly promote the assembly and recruitment of the inflammatory vesicle component of NLRP3 [53, 54]. NLRP3 is an intracellular polyprotein complex consisting of the carrier protein total caspase-1, the adaptor protein ASC, and the stimulus-detecting sensor NLRP3 [55]. Physiologically active caspase-1 is activated by complete caspase-1 upon stimulation of NLRP3 [56, 57]. Caspase-1 activation triggers the inflammatory response by cleaving pro-IL-1β and releasing mature IL-1β [58]. Our findings support the hypothesis that antagonizing P2X7R with BBG, suppresses the expression of NLRP3, caspase-1, and IL-1β in EAN. The alteration of NLRP3 under P2X7R activation or inhibition was confirmed in CD4^+^ T cells both in patients with GBS and EAN models. To the best of our knowledge, this is the first study to provide the evidence that P2X7R and the downstream inflammatory reactions mediated by the NLRP3 inflammasome contribute to EAN.

Additionally, research suggests that P2X7R may control the development of Th1 and Th17 cells via NLRP3 inflammatory activity in CD4^+^T cells [59, 60]. NLRP3 inflammasome assembles in human CD4^ + ^T cells and initiates caspase-1-dependent IL-1β secretion, thereby promoting IFN-γ production and Th1 differentiation in an autocrine fashion [59]. We can’t exclude the potential protective effects of BBG through other cell types beyond CD4^+^T cells, including CD8^+^T cells, macrophages, and Schwann cells, the myelin-producing cells of the peripheral nervous system. Schwann cells play a role in the pathogenesis of GBS by inducing extracellular matrix proteins [61], and membrane damage to Schwann cells can lead to secondary bystander axonal degeneration, resulting in acute axonal injury [62]. P2X7R is reported to be expressed on Schwann cells [63–65]. In our study, we found that P2X7R expression was mildly upregulated in Schwann cells in the EAN model, though the change was not significant (Additional file 1: Fig. S2). P2X7R expression in Schwann cells may also contribute to the alleviation effect of BBG in EAN. Although remains unaltered under EAN induction, the P2X7R expression on CD8^+^T cells and macrophages could also induce a downstream effect under BBG treatment. Further work needs to be conducted in mice conditional knockout of P2X7R in CD4^+^T cells, which might further demonstrate the precise role of CD4^+^T cells and P2X7R/NLRP3 pathway in BBG’s protective effects in GBS.

## Conclusions

Antagonizing P2X7R using BBG can improve EAN symptoms by inhibiting neuroinflammation. This effect is achieved via modulation of Th1 and Th17 differentiation as well as the P2X7R-NLRP3 signaling pathway in CD4^+^T cells. Our findings suggest potential therapeutic strategies based on P2X7R for addressing PNS inflammation.

### Electronic supplementary material

Below is the link to the electronic supplementary material.

Supplementary Material 1

Supplementary Material 2

## Data Availability

All datasets generated and/or analyzed during this study are available from the corresponding author upon reasonable request.

## Abbreviations

GBS: Guillain–Barré syndrome
PNS: Peripheral nervous system
EAN: Experimental autoimmune neuritis
MNCs: Mononuclear cells
BBG: Brilliant Blue G
PBMCs: Peripheral blood mononuclear cells
CMAPs: Compound muscle action potentials
MNCV: Motor nerve conduction velocity
