# Supplementary figures and images for: P2X7 receptor antagonists modulate experimental autoimmune neuritis via regulation of NLRP3 inflammasome activation and Th17 and Th1 cell differentiation
Source: J Neuroinflammation. 2024 Mar 25;21:73. doi: 10.1186/s12974-024-03057-z (PMC10964508; doi:10.1186/s12974-024-03057-z)

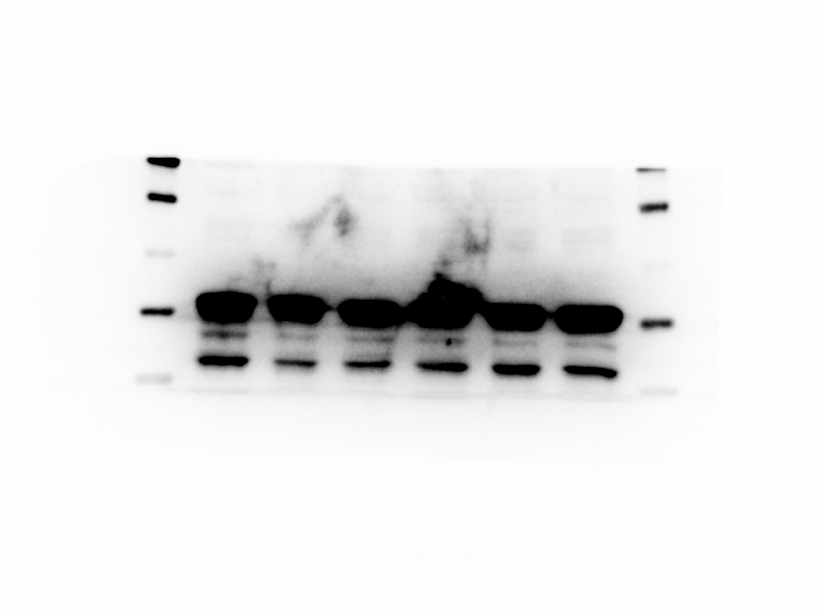


**IL-1β+GAPDH**


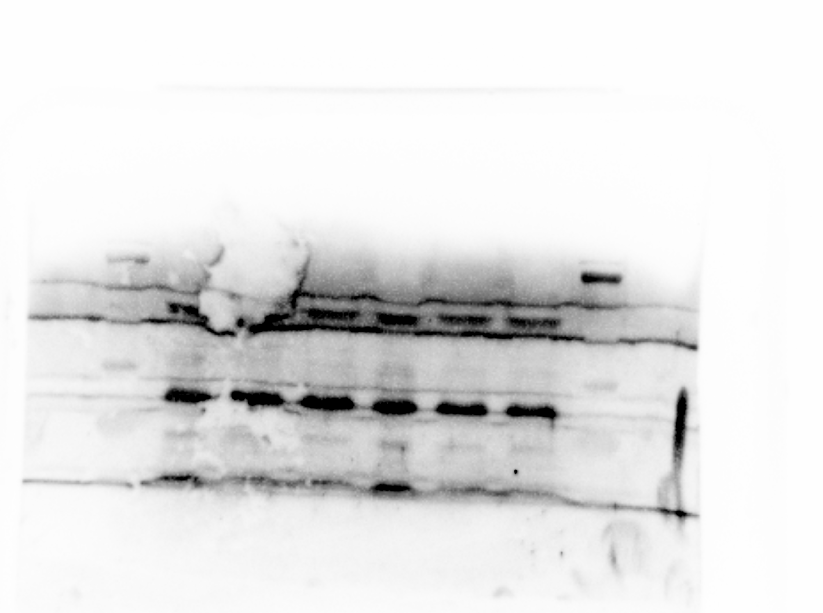


**P2X7+GAPDH**


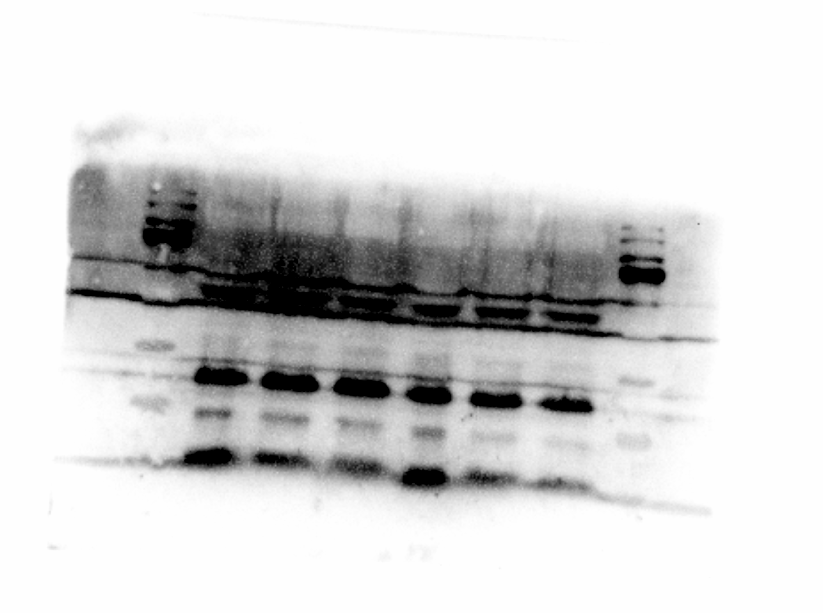


**Caspase-1+GAPDH**


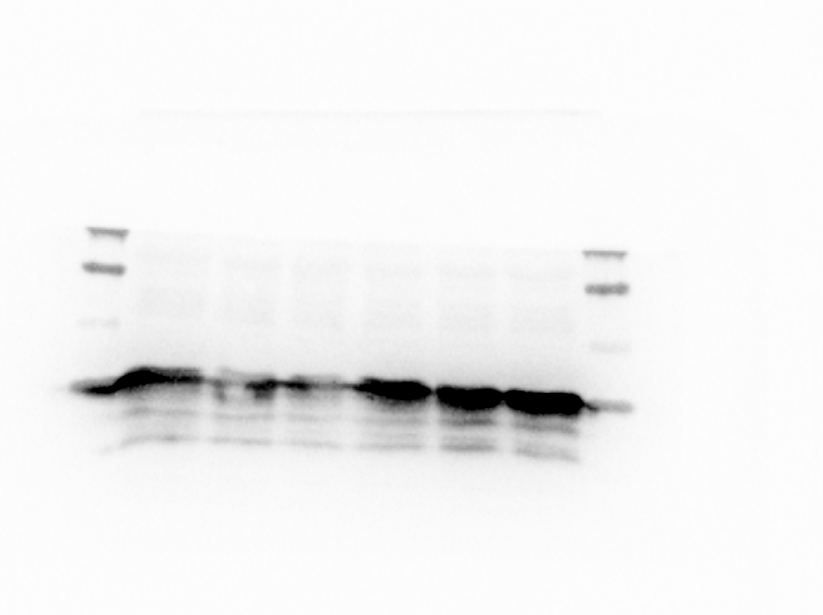


**GAPDH**


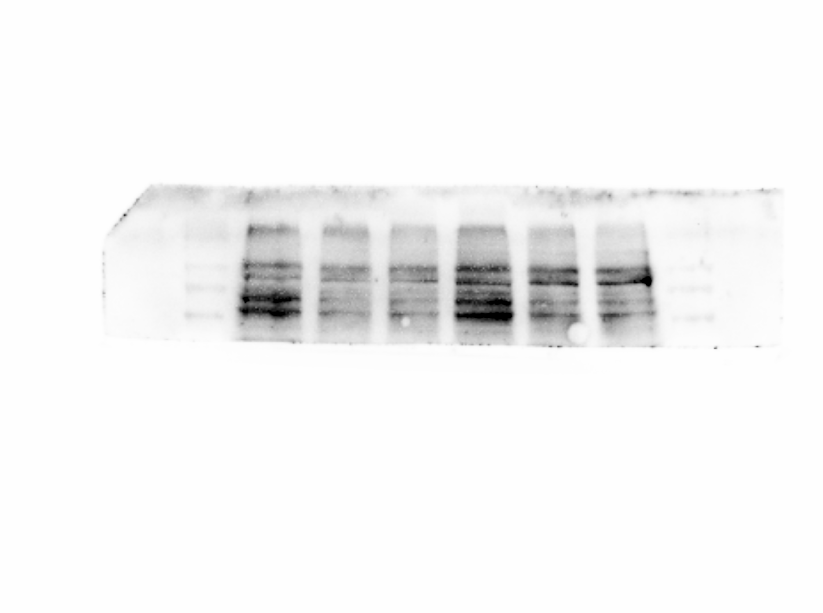


**NLRP3**

Supplement: Supplementary file 1 — Supplementary Material 1 [file 12974_2024_3057_MOESM1_ESM.docx]
