# Supplementary material for: P2X7 receptor antagonists modulate experimental autoimmune neuritis via regulation of NLRP3 inflammasome activation and Th17 and Th1 cell differentiation
Source: J Neuroinflammation. 2024 Mar 25;21:73. doi: 10.1186/s12974-024-03057-z (PMC10964508; doi:10.1186/s12974-024-03057-z)
Supplement: Supplementary file 2 — Supplementary Material 2 [file 12974_2024_3057_MOESM2_ESM.docx]

**Supplementary material**

**P2X7 receptor antagonists modulate experimental autoimmune neuritis via regulation of NLRP3 inflammasome activation and Th17 and Th1 cell differentiation**

Yuhan Xie^1^**^#^**, Ranran Han^2^**^#^**, Yulin Li^2^**^#^**, Weiya Li^1^**^#^**, Shichao Zhang^3^, Yu Wu^1^, Yuexin Zhao^2^, Rongrong Liu^2^, Jie Wu^2^, Wei Jiang^2,4^*, Xiuju Chen^1^*

^1^Department of Neurology, Nankai Hospital, Tianjin Medical University, Tianjin, 300052, China

^2^Department of Neurology, Tianjin Neurological Institute, Tianjin Medical University General Hospital, Tianjin, 300052, China

^3^Department of Interventional Radiology, Zhongshan Hospital of Xiamen University, School of Medicine, Xiamen University, Xiamen 361102, China

^4^Department of Radiology, The Third Affiliated Hospital of Zhengzhou University, Zhengzhou, Henan, China.

**^#^**Y.H.X., R.R.H, Y.L.L. and W.Y.L contributed equally to this work.

*Corresponding author: Prof. Wei Jiang, Email: jiangwei.med@gmail.com

Prof. Xiuju Chen, Email: chyjutj@163.com

**Table S1 Demographics of patients with GBS and healthy controls**

| Characteristic | Real-time quantitative PCR of P2X7R expression | | Flow cytometry | |
| --- | --- | --- | --- | --- |
|  | HCs  (n=8) | GBS  (n=8) | HCs  (n=7) | GBS  (n=7) |
| Male | 2 | 2 | 2 | 2 |
| Age, median (IQR) | 52 (33-59.5) | 53 (30.3-57.3) | 55 (34-67) | 55 (39-64) |
| Disability score at randomisation | | | | |
| Score=3 | n/a | 5 (62.5%) | n/a | 5 (71.4%) |
| Score=4 | n/a | 2 (25.0%) | n/a | 2 (28.6%) |
| Score=5 | n/a | 1 (12.5%) | n/a | 0 (0%) |
| Diarrhoea | n/a | 1 (12.5%) | n/a | 0 (0%) |
| Respiratory tract infection n/a | | 4 (50.0%) | n/a | 4 (57.1%) |

Data are n (%) unless indicated otherwise. All percentages were calculated for patients with available data. HCs, healthy controls; GBS, Guillain–Barre Syndrome.

**Table S2 The primer sequences**

| Gene | | Organism | FORWARD | REVERSE |
| --- | --- | --- | --- | --- |
| IL-17 | | Rat | TGGACTCTGAGCCGCATTGA | GACGCATGGCGGACAATAGA |
| IL-2 | Rat | | CCAAGCAGGCCACAGAATTG | TCCAGCGTCTTCCAAGTGAA |
| TNF-α | Rat | | ATGGGCTCCCTCTCATCAGT | GCTTGGTGGTTTGCTACGAC |
| IFN-γ | Rat | | AGGAACTGGCAAAAGGACGG | TCAGGTGCGATTCGATGACA |
| β-actin | Rat | | ACACCCGCCACCAGTTCG | CCCACGATGGAGGGGAAGAC |
| P2X7R | Homo sapiens | | GACTTCCTCATCGACACTTACTC | TCTTCCTGTAGTAGTATTCGTTGAC |
| β-actin | Homo sapiens | | CATGTACGTTGCTATCCAGGC | CTCCTTAATGTCACGCACGAT |


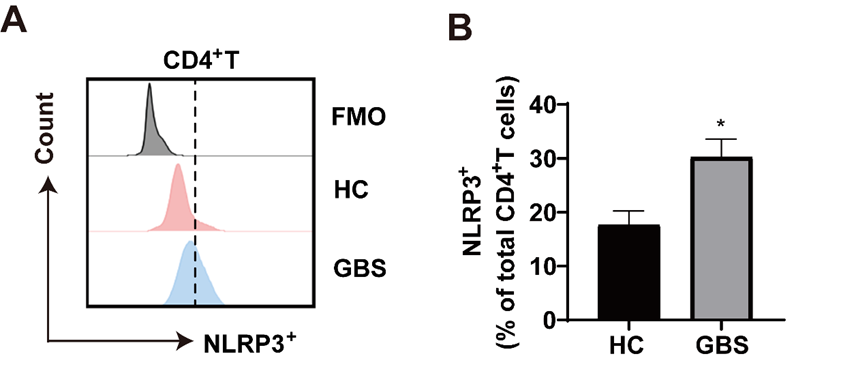


**Figure S1:** NLRP3 expression was examined using flow cytometry. **(A)** Proportions of NLRP3 on CD4^+^ T cells in PBMCs collected from GBS patients and healthy controls. **(B)** Enhanced expression of NLRP3 in CD4^+^ T cells in the peripheral blood collected from GBS patients (n=7 per group). HC, healthy control; GBS, Guillain–Barre Syndrome. **P*<0.05, ***p*<0.01.


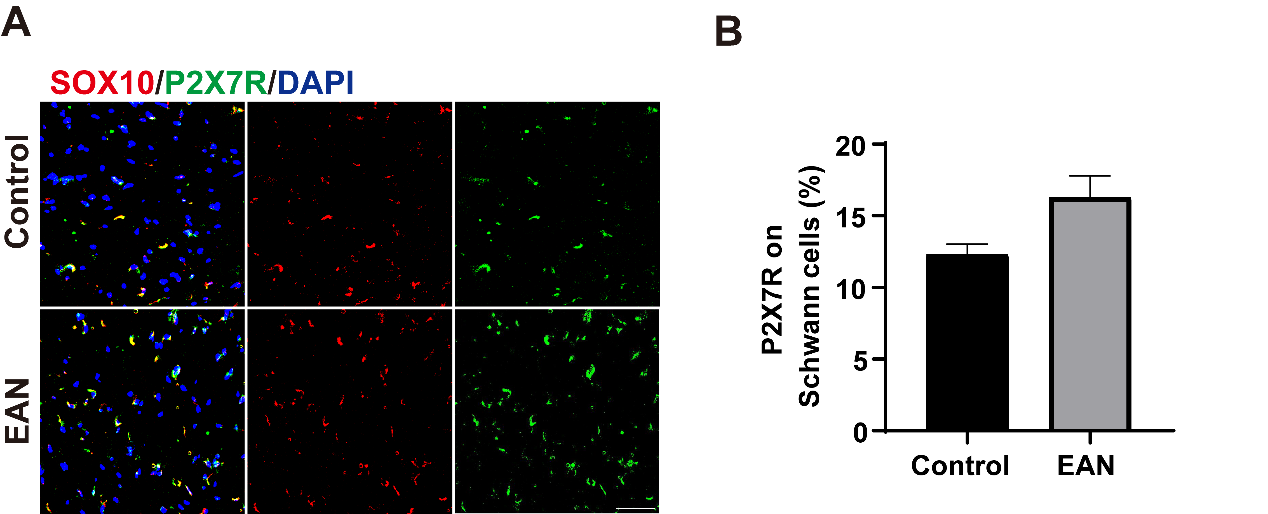


**Figure S2:** The expression of P2X7R in Schwann cells. **(A, B)** Immunofluorescence photomicrographs and quantification of P2X7R expression on Schwann cells (SOX10) in the sciatic nerves of EAN and control (unimmunized group) rats (p=0.0649, Mann–Whitney U test). Scale bars, 20μm.


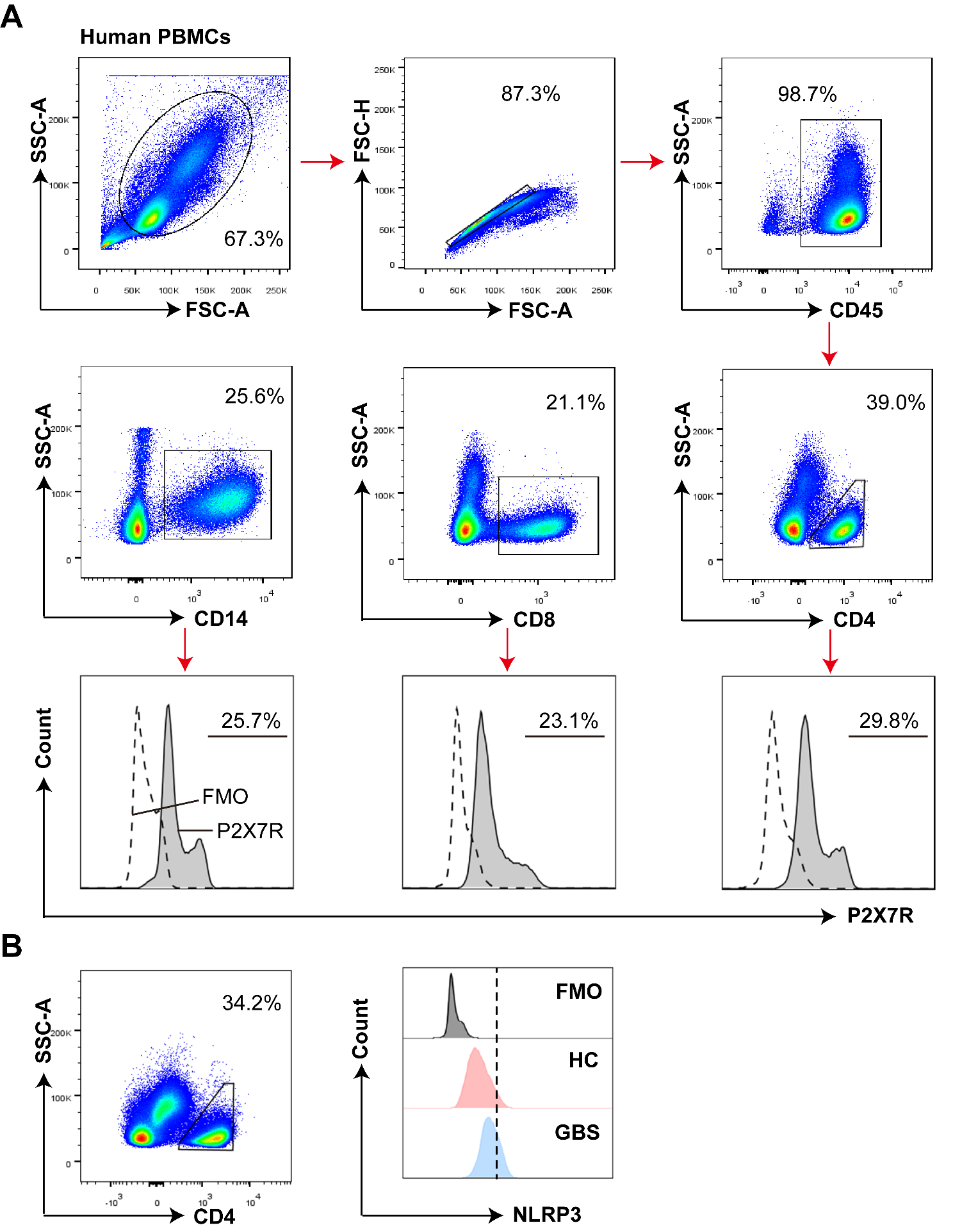


**Figure S3: (A)** Gating strategy of P2X7R in CD4^+^ T cells, CD8^+^ T cells and monocytes in PBMCs by flow cytometry. **(B)** Gating strategy of NLRP3 in CD4^+^ T cells in PBMCs by flow cytometry. A total of 3*10^5^ cells were measured in each sample using flow cytometry.


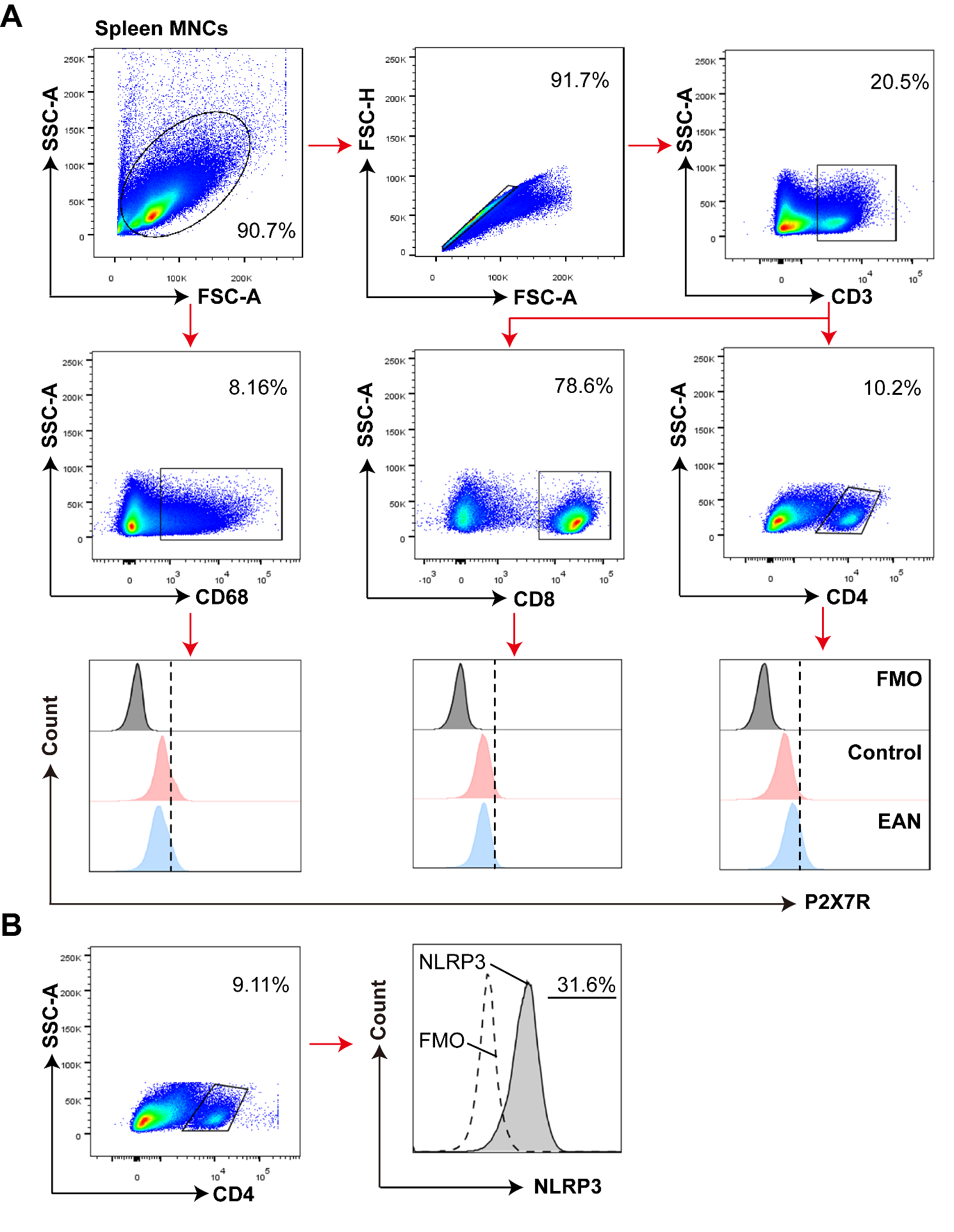


**Figure S4: (A)** Gating strategy of P2X7R in CD4^+^ T cells, CD8^+^ T cells and macrophages in splenic MNCs by flow cytometry. **(B)** Gating strategy of NLRP3 in CD4^+^ T cells in splenic MNCs by flow cytometry. A total of 5*10^5^ cells were measured in each sample using flow cytometry.
